# Water intake, baseline biopsy, and graft function after living donor kidney transplantation

**DOI:** 10.1038/s41598-024-54163-0

**Published:** 2024-02-14

**Authors:** Shigeyoshi Yamanaga, Yuji Hidaka, Chiaki Kawabata, Mariko Toyoda, Kosuke Tanaka, Yasuhiro Yamamoto, Akito Inadome, Asami Takeda, Hiroshi Yokomizo

**Affiliations:** 1 Department of Surgery, Japanese Red Cross Kumamoto Hospital, 2-1-1 Nagamine Minami, Higashi-ku, Kumamoto, 861-8520 Japan; 2 Department of Nephrology, Japanese Red Cross Kumamoto Hospital, Kumamoto, Japan; 3 Department of Surgery, Kyoto University, Kyoto, Japan; 4 Department of Urology, Japanese Red Cross Kumamoto Hospital, Kumamoto, Japan; 5 Department of Nephrology, Japanese Red Cross Nagoya Daini Hospital, Aichi, Japan

**Keywords:** Water intake, Biopsy, Kidney transplantation, Graft function, Medical research, Nephrology, Risk factors, Urology

## Abstract

Increased water intake is recommended for kidney transplant recipients; however, its efficacy remains controversial. We hypothesized that pre-existing histological findings of the allograft might modulate the impact of water intake. We retrospectively analyzed 167 adults with living-donor kidney transplants (April 2011–May 2020; median observation period, 77 months) whose baseline biopsy data were available. We compared the chronic-change group (n = 38) with the control group (n = 129) to assess the impact of self-reported daily water intake on the estimated glomerular filtration rate (eGFR). The range distribution of water intake was as follows: − 1000 ml (n = 4), 1000–1500 ml (n = 23), 1500–2000 ml (n = 64), 2000–2500 ml (n = 57), 2500–3000 ml (n = 16), and 3000 − ml (n = 3). Donor age was significantly higher in the chronic-change group. In the control group, the ΔeGFR/year increase was correlated with water intake. However, the increase in the water intake of the chronic-change group significantly decreased ΔeGFR/year (1000–1500 ml: + 1.95 ml/min/1.73 m^2^ and > 2000 ml: − 1.92 ml/min/1.73 m^2^, p = 0.014). This study suggested a potential influence of increased water intake on recipients with marginal grafts in living donor kidney transplantation.

## Introduction

Active water intake of at least eight glasses per day to improve health is a widely accepted belief that is encouraged in guidelines and social campaigns^1,2^. Increased water intake prevents kidney stones and acute tubular necrosis caused by reduced water intake and heat stress, which would eventually lead to the progression of chronic kidney disease (CKD)^3,4^. Increased water intake theoretically protects against kidney disease^5^ by reducing vasopressin secretion. Vasopressin secretion is related to hyperfiltration-mediated kidney injury^3^, resulting in renal sclerosis^6^. Notably, the total water intake per unit of body weight (BW) is known to decrease with age^7^. To reap survival benefits^8^, increased water intake remains the standard of care following kidney transplantation^5,9^.

Evidence supporting the encouragement of increased water intake is scarce in the literature. Several community-based studies have suggested that increasing water intake positively influences the progression of CKD and the prevention of cardiovascular disease^10,11^. In contrast, CKD population studies have positive^12,13^ and negative^14–16^ results regarding increased water intake (mostly estimated from urine osmolality) in slowing kidney function decline. A recent CKD-REIN cohort study showed that the degree of water intake, the risk of kidney failure, and the decline of kidney function were U-shaped; both decreased (< 1 L) and increased (> 2 L) water intake were not beneficial, suggesting that the optimal range of daily water intake for CKD patients is 1–2 l/day^17^. Due to the concentration deficit from tubular damage and fibrosis, patients with advanced CKD produce low-osmolality urine, which requires an increased water intake, resulting in a rapid estimated glomerular filtration rate (eGFR) decline. In kidney transplantation, no studies have supported or discouraged the impact of increased water intake on graft function^9,18–20^. One possible explanation for these conflicting results is the difference in baseline CKD stages^15^. Furthermore, without specific reasons, CKD patients usually do not undergo kidney biopsies in the clinical setting. CKD staging does not correlate with kidney histological damage, even in different patients with the same eGFR value^21^.

The intensity of tubulointerstitial fibrosis negatively correlates with kidney function in native kidneys^22^. Interstitial fibrosis and tubular atrophy (IFTA) found in baseline biopsies describe the prognosis of long-term outcomes in living and deceased kidney transplantation patients^23–25^. We have also reported that even healthy living donors within the donation criteria^26^ may have pre-existing histopathological damage. This included chronicity change, which is a combination of interstitial fibrosis (ci), tubular atrophy (ct), and arteriolar hyalinosis (ah) of Banff scores^27^ (ci + ct ≥ 1 ∩ ah ≥ 1), which was related to suboptimal compensatory hypertrophy irrespective of donor age^28^. This chronicity change would have a long-term impact on the recipient as well^29^.

To our knowledge, no study, to date, has investigated the impact of water intake and renal biopsy findings on renal function, even in the general and CKD population segments. We hypothesize that the impact of water intake might be affected by the baseline biopsy findings of the allograft in living donor kidney transplantation.

## Results

### Baseline characteristics

The baseline characteristics of the living donors and recipients are presented in Table 1. The median observation period was 77 months (range 46–99 months). There were no significant differences in recipient characteristics, including age, sex, body mass index (BMI), dialysis vintage, preemptive kidney transplantation, and diabetes. In the donor characteristics, age [chronic change (CC) vs. control: 60.0 (54.5–70.0) years old vs. 57.0 (49.0–63.0) years old, p = 0.01], BMI [24.4 (21.6–26.3) kg/m^2^ vs. 22.3 (20.7–24.8) kg/m^2^, p = 0.02], and HbA1c [5.7 (5.5–6.1) % vs. 5.6 (5.4–5.8) %, p = 0.04] were significantly higher in the CC group. Pre-donation eGFR [81.8 (74.1–92.5) ml/min/1.73 m^2^ vs. 80.9 (72.6–91.1) ml/min/1.73 m^2^, p = 0.48] and urine protein (UP) [59 (35–94) mg vs. 50 (34–72) mg, p = 0.20] were not different between the groups. Daily water intake peaked at 1500–2000 ml, and the prevalence did not differ between the groups.

**Table 1:** Baseline preoperative characteristics.

	Overall n = 167	Chronic change n = 38	Control n = 129	P-value
Recipient				
Age, years, median (IQR)	49.0 (36.0–59.0)	48.5 (36.8–54.3)	50.0 (34.0–60.0)	0.56
Sex, male/female, n	115 / 52	24 / 14	91 / 38	0.43
Body weight, kg, median (IQR)	60.6 (52.4–71.0)	59.0 (53.7–73.9)	61.0 (52.1–70.7)	0.87
Height, cm, median (IQR)	166 (160–171)	167.0 (161.8–170.0)	165.7 (159.0–171.9)	0.75
Body mass index, kg/m^2^, median (IQR)	22.2 (19.8–25.1)	21.7 (19.9–25.1)	22.2 (19.8–25.2)	0.79
Dialysis vintage, months, median (IQR)	8.0 (0–37.0)	7.0 (0–27.3)	9.0 (0–41.5)	0.59
Pre-emptive kidney transplantation, n (%)	50 (29.9)	10 (26.3)	40 (31.0)	0.69
Observation period, months, median (IQR)	77.0 (46.0–99.0)	71.5 (52.0–91.5)	80.0 (46.0–100.0)	0.54
Diabetic nephropathy, n (%)	30 (18.0)	4 (10.5)	26 (20.2)	0.23
Daily water intake				
– 1000 ml	4 (2.4%)	1 (2.6%)	3 (2.3%)	0.84
1000–1500 ml	23 (13.8%)	6 (15.8%)	17 (13.2%)	
1500–2000 ml	64 (38.3%)	17 (44.7%)	47 (36.4%)	
2000–2500 ml	57 (34.1%)	11 (28.9%)	46 (35.7%)	
2500–3000 ml	16 (9.6%)	3 (7.9%)	13 (10.1%)	
3000 – ml	3 (1.8%)	0 (0%)	3 (2.3%)	
Donor				
Age, years, median (IQR)	58.0 (51.0–65.0)	60.0 (54.5–70.0)	57.0 (49.0–63.0)	0.01
Sex, male/female	60 vs 107	18 vs 20	42 vs 87	0.84
Body weight, kg, median (IQR)	58.3 (52.0–66.1)	62.5 (51.7–72.6)	57.0 (52.0–65.0)	0.07
Height, cm, median (IQR)	159.1 (154.0–166.0)	159.1 (153.9–168.9)	159.5 (154.5–165.0)	0.81
Body mass index, kg/m^2^, median (IQR)	22.7 (20.8–25.1)	24.4 (21.6–26.3)	22.3 (20.7–24.8)	0.02
Serum creatinine, mg/dl, median (IQR)	0.65 (0.56–0.73)	0.66 (0.57–0.72)	0.64 (0.55–0.74)	0.51
eGFR, ml/min/1.73 m^2^ median (IQR)	81.0 (72.7–91.4)	81.8 (74.1–92.5)	80.9 (72.6–91.1)	0.48
CCr, ml/min, median (IQR)	105.9 (95.1–120.4)	104.4 (89.6–119.7)	106.4 (96.4–120.6)	0.38
Measured GFR, ml/min/1.73 m^2^, median (IQR)	115.4 (101.2–133.0)	110.2 (99.5–136.0)	116.2 (101.7–133.0)	0.60
Urine protein, mg, median (IQR)	51.0 (34.0–78.0)	59.0 (35.0–94.0)	50.0 (34.0–72.0)	0.20
LDL-C, mg/dl, median (IQR)	118.0 (99.5–140.5)	117.5 (98.0–134.3)	119.0 (101.0–145.0)	0.49
HbA1c, %, median (IQR)	5.6 (5.4–5.9)	5.7 (5.5–6.1)	5.6 (5.4–5.8)	0.04
ABO blood type incompatible	48 (28.7%)	10 (26.3%)	38 (29.5%)	0.84
Pre-DSA	14 (8.4%)	4 (10.5%)	10 (7.8%)	0.53

*CCr* creatinine clearance, *DSA* donor specific antibody, *eGFR* estimated glomerular filtration rate, *IQR* interquartile range, *LDL-C* low-density lipoprotein cholesterol.

### Graft and patient outcomes

The graft and patient outcomes are shown in Table 2. Five-year graft and patient survival rates did not differ between the groups. The rates of acute rejection and de novo donor-specific antibody (DSA) did not differ between groups. Although serum creatinine (S-Cr) and eGFR at 1 year and the latest year were significantly lower in the CC group, ΔeGFR/year between the groups was not significantly different [− 0.59 (− 1.77 to 0.59) ml/min/1.73 m^2^/year vs. − 0.70 (− 1.93 to  + 0.74) ml/min/1.73 m^2^/year, p = 0.74], and the decline in eGFR did not depend on eGFR at 1 year.

**Table 2:** Graft and patient outcomes.

	Overall n = 167	Chronic change n = 38	Control n = 129	P-value
Five-year graft survival, %	95.5	97.4	93.7	0.92
Five-year patient survival, %	99.1	100.0	98.9	0.56
S-Cr at 1 year post-Tx, mg/dl, median (IQR)	1.27 (1.00–1.46)	1.40 (1.11–1.65)	1.20 (1.00–1.43)	0.017
S-Cr at the latest year post-Tx, mg/dl, median (IQR)	1.25 (1.06–1.53)	1.40 (1.11–1.77)	1.24 (1.04–1.46)	0.038
eGFR at 1 year post-Tx, ml/min/1.73 m^2^, median (IQR)	47.0 (39.4–54.0)	41.8 (34.0–50.6)	48.8 (41.4–56.1)	0.004
eGFR at the latest year post-Tx, ml/min/1.73 m^2^, median (IQR)	44.0 (36.3–52.9)	38.7 (32.4–51.0)	45.2 (39.1–53.1)	0.007
ΔeGFR/year, ml/min/1.73 m^2^/year, median (IQR)	− 0.7 (− 1.9 ~ + 0.56)	− 0.59 (− 1.77 ~ + 0.59)	− 0.70 (− 1.93 ~ + 0.74)	0.740
CCr at 1 year, ml/min, median (IQR)	61.5 (50.8–71.5)	59.3 (48.3–67.5)	61.6 (51.0–72.2)	0.21
CCr at latest year, ml/min, median (IQR)	53.5 (42.2–68.5)	47.9 (35.9–65.5)	55.5 (43.7–69.0)	0.27
Urine protein at 1 year, mg, median (IQR)	135.0 (70.0–252.0)	154.5 (70.0–395.0)	127.0 (69.0–226.0)	0.17
Urine protein at the latest year, mg, median (IQR)	136.0 (63.0–277.0)	180.0 (99.5–493.5)	123.5 (61.8–263.3)	0.07
Urine NaCI /day at 1 year, g, median (IQR)	8.5 (6.5–10.9)	8.6 (6.8–11.8)	8.5 (6.4–10.9)	0.68
Urine NaCI /day at the latest year, g, median (IQR)	7.9 (5.5–9.9)	7.5 (4.6–10.5)	8.0 (5.7–9.7)	0.73
Non-adherence, n (%)	32 (19.2%)	8 (21.1%)	24 (18.6%)	0.82
Acute rejection, n (%)	15 (9.0%)	3 (7.9%)	12 (9.3%)	1.00
De novo DSA, n (%)	23 (13.8%)	8 (21.1%)	15 (11.6%)	0.18
Body weight change/year, kg, median (IQR)	+ 2.7 (− 0.6 ~ + 7.0)	+ 2.2 (− 3.4 ~ + 6.8)	+ 3.0 (− 0.3 ~ + 7.0)	0.26

*CCr* creatinine clearance, *DSA* donor-specific antibody, *eGFR* estimated glomerular filtration rate, *IQR* interquartile range, *LDL-C* low-density lipoprotein cholesterol, *S-Cr* serum creatinine, *Tx* transplantation.

### The impact of water intake on graft function and survival

**Figure 1:**
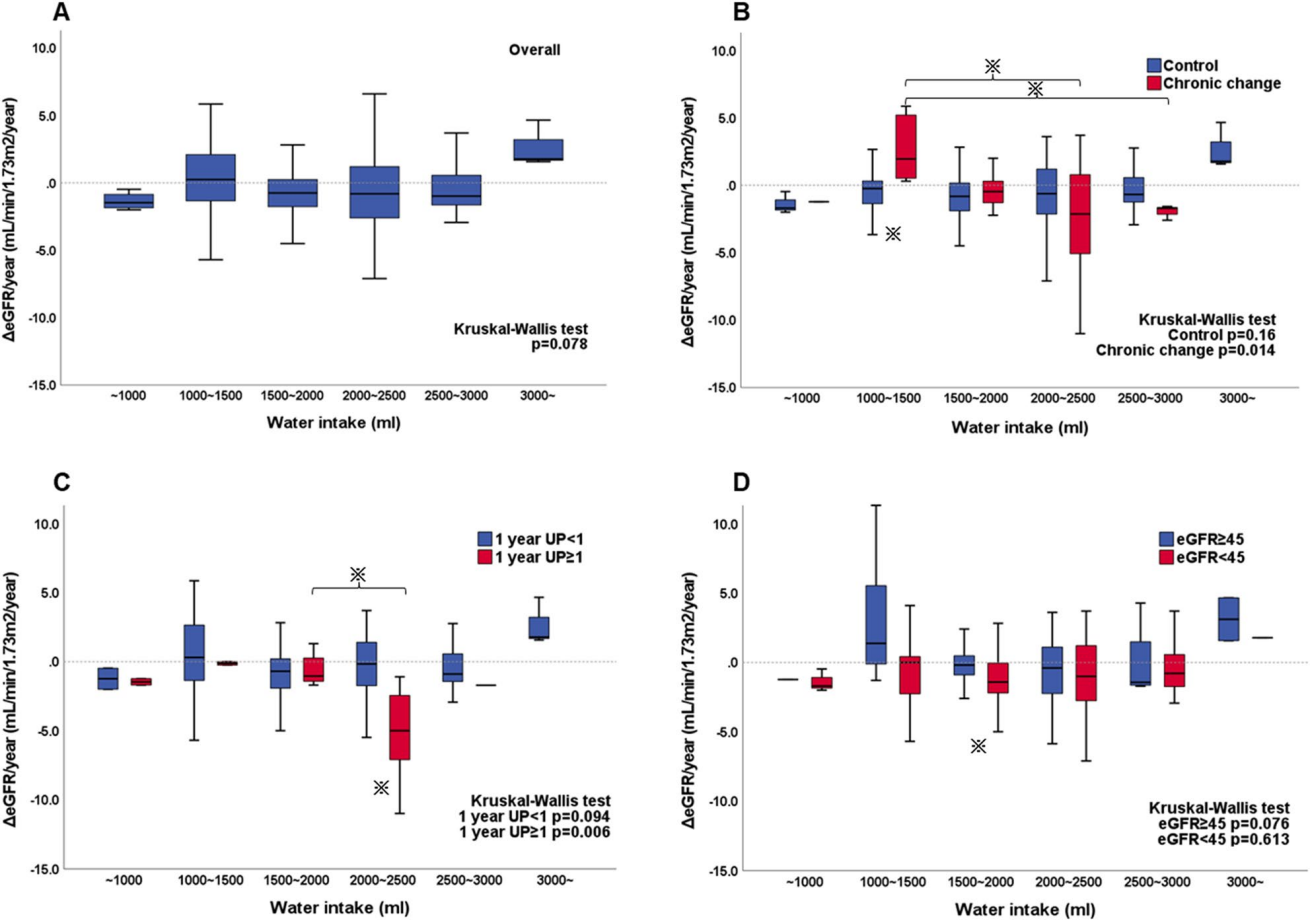
The relationship between the ΔeGFR/year and the amount of daily water intake. (**A**) Overall, (**B**) control vs. chronic change group, (**C**) 1-year UP < 1 vs. 1-year UP ≥ 1, and (**D**) 1-year eGFR < 45 vs. 1-year eGFR ≥ 45. ^※^p < 0.05. *eGFR* estimated glomerular filtration rate, *ΔeGFR/year* eGFR slope, *UP* urine protein.

**Figure 2:**
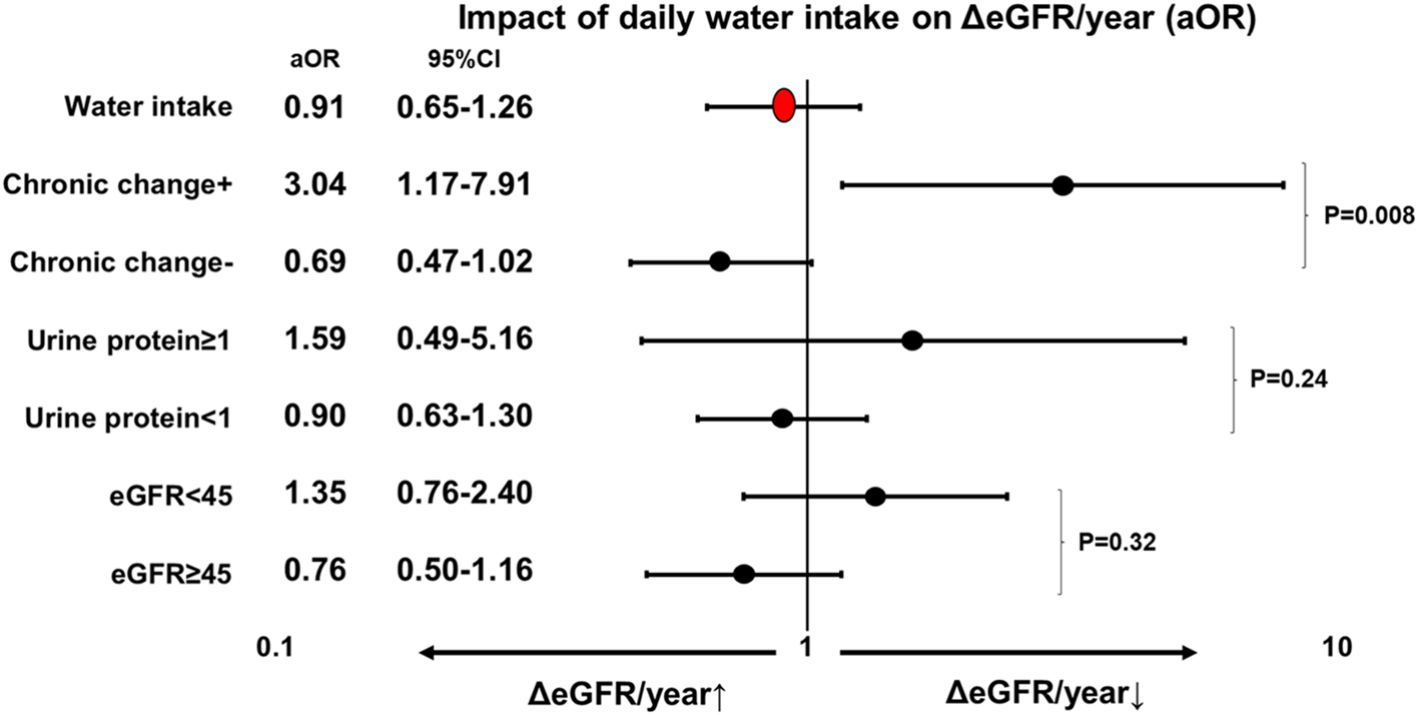
Interaction analyses for the impact of daily water intake on the ΔeGFR/year. The odds ratio for ΔeGFR/year < − 1 or not. Adjusted for recipient gender and age. P values for interactions. *aOR* adjusted odds ratio, *CI* confidence interval, *eGFR* estimated glomerular filtration rate, *ΔeGFR/year* eGFR slope.

**Figure 3:**
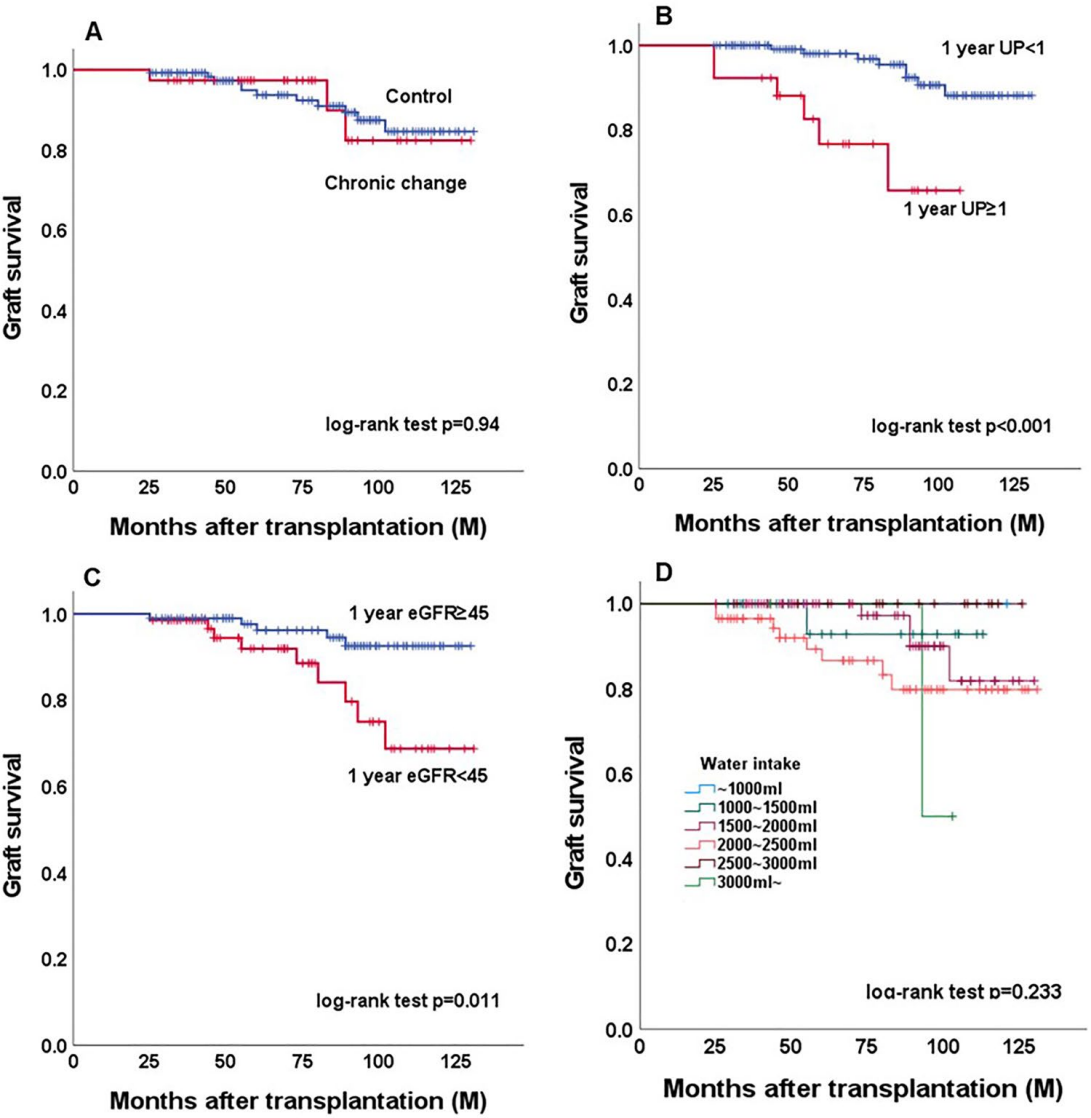
Graft survivals after living donor kidney transplantation. (**A**) Control vs. chronic change group, (**B**) 1-year UP < 1 vs. 1-year UP ≥ 1, (**C**) 1-year eGFR ≥ 45 vs. 1-year eGFR < 45, and (**D**) water intake category. *eGFR* estimated glomerular filtration rate, *UP* urine protein.

Overall, increased water intake was positively correlated with the eGFR slope (Fig. 1A, p = 0.078). However, in the CC group (Fig. 1B), ΔeGFR/year declined significantly as water intake increased (p = 0.014); peaked at 1000–1500 ml [+ 1.95 (+ 0.46– + 5.36)], and declined thereafter [> 2000 ml, − 1.92 (− 4.66 to + 0.49) ml/min/1.73 m^2^/year]. One-year UP ≥ 1 seemed to have a similar impact as CC, while 1 year-eGFR < 45 did not (Fig. 1C,D). In the sensitivity analysis, the impact of water intake on graft function (declining more than 1 ml/min/1.73 m^2^/year) showed an association in the CC group but not in the UP ≥ 1 or eGFR < 45 subgroups (Fig. 2). In the survival analysis, CC and water intake did not impact overall survival. However, UP ≥ 1 and eGFR < 45 did (Fig. 3).

### Post-hoc sample size calculation

A post-hoc sample size calculation was performed to determine the minimum sample size needed to achieve reliable results for this cohort. We set a margin of error rate, confidence level, population proportion, and population size of 5%, 95%, 22.8%, and n = 167, respectively. The cohort size in this study comfortably exceeded the calculated minimum sample size requirement of n = 104.

## Discussion

In this study, we have proposed a potential association between increased water intake and the slope of kidney function based on pre-existing pathological status in living donor kidney transplantation. The strength of the present study is that we could clarify the baseline status by drawing a comparison with baseline biopsy, UP ≥ 1^30^, and eGFR < 45^31^. Furthermore, our study further confirmed that pre-donation eGFRs were not correlated with baseline biopsy findings in the otherwise healthy living donor population.

Notably, increased water intake differed according to the baseline histological changes; in the CC group, graft function was best preserved or improved with 1000–1500 ml daily water intake and decreased thereafter. The relationship between water intake in the CC group and the eGFR slope was U-shaped in our study, which was in line with the CKD-REIN cohort study^17^. As per the present study, 1000–2000 ml water intake per day could be recommended, irrespective of the baseline biopsy status. However, a further increase in water intake improved the eGFR slope in the control group. The positive impact of increased water intake was also observed in the UP < 1 and eGFR ≥ 45 groups. As expected, the CC group had a higher proportion of older donors, higher BMI, and higher HbA1c levels at baseline than the control group. However, this did not directly impact the distribution of daily water intake categories, as it remained normally distributed in both of the two groups.

The impact of this increased water intake on graft function might differ between healthy single kidneys and pre-diseased single kidneys. In healthy 5/6 nephrectomized rats, increased water intake effectively slowed the progression of early chronic renal failure [increased creatinine clearance (CCr), reduced proteinuria, hypertrophy, sclerosis, and fibrosis] by reducing vasopressin^32,33^. It failed to increase CCr in a streptozotocin-induced diabetic rat model^34^, possibly because of the AVP-resistant downregulation of aquaporin receptors^35^. Increased plasma flow due to increased water intake for a pre-diseased single kidney might induce glomerular hyperfiltration, resulting in hypertrophy of the allograft and finally leading to sclerosis^36^. Furthermore, increased water intake might induce “renal tamponade” or compression of the kidney in fibrotic kidneys^37^. Reduced elastosis in the transplanted, ectopic kidney would be unable to withstand the micro-intestinal edema as there is limited space for pressure escape, resulting in tubular compression and ischemia^37^. However, the concept of renal tamponade has not been established in transplanted kidneys, it has been mainly used in patients with fluid overload and cardiac failure^37,38^.

In the clinical setting, general population studies consistently report the benefit of increased water intake in lowering CKD prevalence and slowing the kidney function slope. However, discrepancies exist in the CKD population regarding increased water intake^12–14,16–20^, which might arise from the different baseline stages^15^, since stage evaluations were solely dependent on eGFR. The varied effects of water intake depending on the baseline pathological status of the kidney in this study would implicate a new target for novel CKD studies and management. Notably, adherence to NaCl restriction was slightly better in the CC group. Considering there was no difference in the recipient characteristics between the CC and control groups, informing recipients of the baseline biopsy result might have affected their attitude toward a better lifestyle and improved graft survival^39^.

There are several limitations that should be considered alongside the results of this study. Because of the single-center retrospective design and the constrained sample size, the exclusion of potential confounders was not feasible, and a detailed analysis within the chronic change (CC) group could not be undertaken. Due to the substantial dissimilarities in characteristics between recipients and donors, employing statistically robust adjustments or matching methods to mitigate potential confounding factors was challenging. Besides the baseline biopsy, we did not regularly perform protocol biopsies. Therefore, it would be of great interest if we could analyze patient data with respect to the progression of fibrosis, atrophy, and sclerosis after kidney transplantation. We did not include the possible impact of tacrolimus (TAC) or cyclosporine on the chronic damage and deterioration of kidney function. We acknowledge that the reliability of self-reporting water intake is limited. Prospective studies employing alternative methodologies, such as "smart" water bottles, represent the forthcoming challenge aimed at enhancing the precision of estimation^40^. Although we employed arbitrary water intake categorization, considering the measurement of fluid requirements using objective parameters, such as bioimpedance, for future research is advisable since this could provide a more accurate assessment^41^. Care should be taken in interpreting the implications of increased water intake and eGFR decline in the chronic change group, given the potential influence of confounding factors such as healthcare providers' instructions to increase water intake. Furthermore, the disparity between eGFR decline and graft survival could stem from the distinct time points of measurements, with baseline biopsy conducted at the time of implantation and urine protein/eGFR assessments performed at 1-year after transplantation. Further prospective studies are essential for a more comprehensive understanding of the relationship between chronic pathological changes and water intake in graft function after living donor kidney transplantation.

In conclusion, this study suggested a potential influence of increased water intake on recipients with marginal grafts in living donor kidney transplantation. Further studies are needed to confirm the effects of increased water intake and pathological findings in CKD and kidney transplant patients.

## Methods

Our institution conducted 189 consecutive living donor kidney transplantations from April 2011 to May 2020. Among these, 5, 2, 6, and 9 cases with graft failure within 2 years, with unavailable baseline biopsy data, involving pediatric patients (aged < 18 years at the time of transplantation), and with lost follow-up or incomplete data, respectively, were excluded from the analysis. Eventually, 167 cases were included in this retrospective analysis. To examine the effect of self-reported daily water intake on ΔeGFR/year according to baseline biopsy findings, the CC group (IFTA or chronicity change defined as ct + ci ≥ 1 ∩ ah ≥ 1, n = 38) was compared with the control group (n = 129). The next section of this manuscript describes a detailed definition of the CC group of patients.

The corresponding data were collected from electronic medical records. The recipient characteristics were as follows: age, gender, BW, (kg), height, BMI (kg/m^2^), dialysis vintage, presence of diabetic nephropathy, observation period, pre-emptive kidney transplant, S-Cr, eGFR, UP, ABO blood type incompatibility, and pre-existing DSA. The preoperative donor characteristics were as follows: age, gender, BW, BMI, height, S-Cr, eGFR, CCr, measured GFR, UP, low-density lipoprotein cholesterol, and HbA1c. All donors were selected by the Japanese donor selection criteria^26^. The standard immunosuppressive regimen included TAC, mycophenolate mofetil (MMF), and methylprednisolone. The desensitization therapy consisted of rituximab, plasmapheresis, and MMF. The eGFR slope (ΔeGFR/year) was calculated (latest eGFR − 1-year post-transplant eGFR)/(postoperative years of the latest eGFR − 1). Nonadherence was defined as whether the recipients had a prior history of skipping scheduled outpatient visits. All acute rejections (including T cell-mediated and antibody-mediated rejection) were biopsy-proven. De novo DSA (A, B, Cw, DR, DQ) was defined as more than 1000 mean fluorescence intensities measured by single human leukocyte antigen (HLA)-coated synthetic flow bead analysis (LABScreen^®^ Single Antigen HLA Class I and II, One Lambda, CA, USA).

This study was approved by the institutional review board of the Japanese Red Cross Kumamoto Hospital (Approval Number 513). The review board waived the requirement for informed consent based on the nature of this research. None of the transplant donors were from a vulnerable population, and all donors or their next of kin provided written informed consent. The clinical and research activities being reported are consistent with the Principles of the Declaration of Istanbul as outlined in the 'Declaration of Istanbul on Organ Trafficking and Transplant Tourism' and in accordance with the principles of the Declaration of Helsinki.

### Water intake data collection

Data on water intake were collected from the medical notes that all patients were required to record (water intake, urine output, and physical activity) at every outpatient visit to our institution. The recipients were instructed to note only plain water intake. Considering the seasons and work environments, water intake ranges were categorized as – 1000, 1000–1500, 1500–2000, 2000–2500, 2500–3000, and 3000 – ml.

### Pathological diagnosis

Baseline biopsy was done 1 h after reperfusion during the transplantation. Data were collected from the pathological reports. No protocol biopsies using other timings were included in this study. Chronic histopathological findings were classified according to the banff classification as ci, ct, and ah^27^. Other than IFTA, the definition of chronicity change is the combination of those above (ci + ct ≥ 1 ∩ ah ≥ 1) as previously reported^28,29^.

### Statistical analysis

Variables were analyzed using Chi-square and Fisher’s exact tests for categorical data and Mann–Whitney U and Kruskal–Wallis H test tests for continuous data. Unless otherwise specified, all continuous data are expressed as medians (interquartile range) considering the non-parametric distribution. Multivariate analysis was used to adjust for the effect of water intake on graft function. Survival analyses were performed using the Kaplan–Meier method, and statistical differences between the curves were assessed using the log-rank test. A post-hoc sample size calculation was conducted to determine the minimum sample size for this cohort using the Sample Size Calculator (https://www.calculator.net/sample-size-calculator.html). Statistical significance was set at p < 0.05. For multiple comparisons, Bonferroni correction was applied to correct false-positive results. IBM SPSS Statistics for Windows, version 28 (IBM Corp., Armonk, NY, USA) was used for statistical analysis.

## Data Availability

Data are available upon request from the corresponding author, S.Y.
